# Consumer Nutrition Environments of Hospitals: An Exploratory Analysis Using the Hospital Nutrition Environment Scan for Cafeterias, Vending Machines, and Gift Shops, 2012

**DOI:** 10.5888/pcd10.120335

**Published:** 2013-07-03

**Authors:** Courtney P. Winston, James F. Sallis, Michael D. Swartz, Deanna M. Hoelscher, Melissa F. Peskin

**Affiliations:** Author Affiliations: James F. Sallis, Division of Behavioral Medicine, University of California, San Diego, and Active Living Research, San Diego, California; Michael D. Swartz, The University of Texas Health Science Center at Houston, Houston, Texas; Deanna M. Hoelscher, Michael & Susan Dell Center for Healthy Living and University of Texas School of Public Health, Austin, Texas; Melissa F. Peskin, University of Texas Prevention Research Center and University of Texas Health Science Center School of Public Health, Houston, Texas.

## Abstract

**Introduction:**

Hospitals are the primary worksite of over 5 million adults in the United States, and millions of meals are procured and consumed in this setting. Because many worksite nutrition initiatives use an ecological framework to improve the dietary habits of employees, the nutrition values of foods served in hospitals is receiving attention.

**Methods:**

This study used the Hospital Nutrition Environment Scan for Cafeterias, Vending Machines, and Gift Shops to quantitatively describe the consumer nutrition environments of 39 hospitals in Southern California. Data were collected by visiting each facility once from February 2012 through May 2012.

**Results:**

On average, hospitals achieved only 29%, 33%, and less than 1% of the total possible points for their cafeteria, vending machines, and gift shops sections, respectively; overall, hospitals scored 25% of the total possible points. Large facility size and contracted food service operations were associated with some healthy practices in hospital cafeterias, but we found no association between these variables and the sectional or overall nutrition composite scores.

**Conclusion:**

The average consumer nutrition environment of hospitals in this sample was minimally conducive to healthful eating. Nutrition-related interventions are warranted in hospital settings.

## Introduction

Public health practitioners use science-based theoretical models to guide the development of behavior change interventions (1–3). When crafting interventions for complex behaviors such as dietary intake, individual-level models may not address all behavioral antecedents as effectively as comprehensive, multilevel approaches (4). The ecological framework, a model recommended for nutrition-related interventions, is one comprehensive approach, and it posits 4 levels of influence on dietary behaviors: individual factors, social environments, physical environments, and macro-level environments (5). Over the past decade, one of the 4 levels of the ecological model, the physical environment, has become a major target for nutrition-related interventions (6,7).

Four physical environments (consumer nutrition environment, organizational nutrition environment, community nutrition environment and information environment) have been described (6) that align with Story et al’s ecological framework (5). These 4 environments collectively describe the greater nutrition environment. Government and organizational policies influence these 4 environments which, in turn, may also affect eating behaviors directly.

The consumer nutrition environment describes what a person encounters when visiting a food service outlet. The consumer nutrition environment can be captured by observing what healthful food choices are available, comparing price differentials between healthful and unhealthful foods, and describing the placement and promotion of healthful foods. Consumer nutrition environments of commonly accessed food outlets have previously been described by using tools such as the Nutrition Environment Measures Survey for Restaurants (NEMS-R), for Stores (NEMS-S) and for Vending (NEMS-V); all of these tools have demonstrated acceptable psychometric properties (8–10).

Although restaurants, stores, and vending machines are common food service outlets for community members, worksite cafeterias, vending machines, and snack shops are common food service outlets for working adults. Because more than 5 million adults currently work in US hospitals (11), the consumer nutrition environment of this setting could play a major role in influencing the diets of a large population of US adults.

The consumer nutrition environment of hospitals is unique in that it is typically composed of multiple food outlets (eg, cafeteria, vending machines, gift shops), and in some hospitals, each outlet may be managed by a different hospital department or contracted company. Each of these outlets plays a critical role in determining the overall consumer nutrition environment, and each operation should be assessed when using an ecological approach for workplace interventions related to the consumer nutrition environment.

In 2011 our research team adapted a comprehensive measurement tool for assessing the consumer nutrition environment of hospital settings. The outcome of this collaboration was the Hospital Nutrition Environment Scan for Cafeterias, Vending Machines, and Gift Shops (HNES). After rigorous scientific testing, this tool was found to have acceptable reliability metrics (12). Given the emerging interest in improving hospitals’ consumer nutrition environments (13–18), HNES has several implications for public health, health care systems, and worksite wellness.

Our study used HNES to quantitatively describe the current state of consumer nutrition environments (ie, cafeterias, vending machines, and gift shops) in a diverse sample of 39 Southern California hospitals. Prevalence estimates were used to determine the percentage of hospitals engaging in healthful nutrition practices and to specify which practices were most and least commonly implemented. The study also examined associations among recommended nutrition practices, hospital facility size, and the type of management used in food service operations (ie, internally operated vs contracted food service management). We hypothesized that larger facilities would have more financial and human resources to assist with creating a good consumer nutrition environment. Likewise, we hypothesized that cafeterias with contracted food service operations would have higher nutrition composite scores, because contracted companies have more regional and national resources than smaller, internally managed food service departments. Also, contracts with outside providers could possibly include specifications for better healthfulness or nutrition content of food offerings.

Associations between hospital nutrition composite scores and socioeconomic characteristics of the hospital neighborhoods were also explored in this study. Previous studies of nutrition environments have suggested a link between neighborhood socioeconomic indicators and the availability of healthful foods (19,20). However, such associations have not been explored between hospital neighborhoods and the hospital nutrition environment. We hypothesized that hospitals in census tracts with high socioeconomic status would have higher nutrition composite scores because they serve more affluent populations who may expect or demand more healthful foods.

## Methods

### Study design and sample

This study was a cross-sectional, descriptive survey of acute-care hospitals in Southern California: San Diego, Los Angeles, San Bernadino, Orange, and Riverside counties. A convenience sample of 39 hospitals was surveyed, and hospitals were primarily selected on the basis of their proximity to research staff and other hospitals and geographic locale. Only hospitals with Joint Commission accreditation were included. Although the hospitals constituted a convenience sample, they represented a variety of community locales (eg, suburban, urban, rural), hospital types (eg, academic/teaching, community, tertiary), and hospital sizes (eg, licensed bed count, number of employees).

### Hospital Nutrition Environment Scan for Cafeterias, Vending Machines, and Gift Shops survey

Adapted from NEMS and developed in collaboration with multiple public health agencies and partners, HNES is a nutrition environment survey that has demonstrated face and content validity. The scan includes sections that assess 3 common hospital food service outlets: cafeterias, vending machines, and gift shops. HNES demonstrated good inter-rater reliability with 74% to 100% interobserver agreement, κ statistics of 0.48 to 1.0 for individual questions, and an intraclass correlation coefficient of 0.961 for the overall nutrition composite score (12).

Each section of HNES asks a variety of environmental questions that can be answered by single observation. Sections begin with questions pertaining to facilitators of and barriers to healthful eating (eg, “Are there signs or displays that encourage healthful eating?”). Each section also contains questions on pricing strategies (eg, pricing differentials for healthful vs unhealthful items) and on advertising promotions for healthful foods and beverages. HNES also assessed the healthfulness of foods and beverages at the point of purchase and calculated the percentages of shelf space for healthful items. HNES criteria for healthful foods and beverages are congruent with criteria used in other NEMS surveys (8–10).

Questions about the consumer nutrition environment are weighted according to their relevance to nutrition environment constructs and are based on points derived from the observed environments. A nutrition composite score is calculated for the 3 sections of HNES. The overall nutrition composite score for the hospital is the sum of scores for all 3 sections: cafeteria, vending machines, and gift shops. The maximum overall score is 177 points, and the maximum section scores are 85, 60, and 32 points for the cafeteria, vending machines, and gift shop sections, respectively.

### Data collection

All hospitals were surveyed from February through May 2012, and during the survey, data were collected by raters who were fully trained on HNES. Rater training consisted of individual instruction, field practice, and review of practice observations. Demographic information for each hospital (eg, licensed bed-count, facility type, number of employees) was gathered from credible Internet resources (eg, the Joint Commission’s website) or from personal communication with a hospital administrator.

### Data analysis

All data analyses were conducted by using SPSS Statistics 18.0 (IBM Corp, Chicago, Illinois). For each section (cafeteria, vending machine, gift shop), HNES results were interpreted by descriptive statistics (ie, mean, standard deviation, minimum and maximum) for the continuous variables and by frequency distribution for the categorical variables. To detect differences among groups, we performed *t* tests, χ^2^, and Fisher’s exact tests for continuous and categorical data to detect differences among the 3 sections (cafeteria, vending, gift shop). To evaluate the relationship between nutrition composite scores and socioeconomic indicators of the census tract in which the hospital is located, normality was assessed and Spearman’s rank correlation coefficients were calculated. Post hoc tests were performed to account for multiple comparisons. All significance levels were set at *P* <.05. The University of Texas Health Science Center Committee for the Protection of Human Subjects exempted this study from internal review board approval given that no human subjects were involved and that all data collected were observed in publicly available spaces.

## Results

About half of the hospitals surveyed in this study were located in an urban area, and about half were in a census tract in which median annual household income was below $60,000 per year (Table 1). Two-thirds of the facilities were considered general or community hospitals, and the remainder were classified as tertiary care, specialty, psychiatric or “other.” Two-thirds of the hospitals had 1,000 to 5,000 employees, and most hospitals had fewer than 500 licensed beds.

**Table 1:** Demographic Characteristics of Hospitals Surveyed and Their Corresponding Neighborhoods (N = 39)

Characteristics	n (%)
**Locale**	
Urban	19 (48.7)
Suburban/Rural	20 (51.3)
**Hospital census tract socioeconomic status indicators**	
Median household income, $	
<60,000	20 (51.3)
>60,000	19 (48.7)
Education, adults aged >25 yr with college education	
<50%	28 (71.8)
>50%	11 (28.2)
**Hospital type**	
Tertiary	4 (10.3)
General/community	26 (66.7)
Specialty (eg, surgical, children’s)	5 (12.8)
Psychiatric	2 (5.1)
Other	2 (5.1)
**Number of employees**	
<,1,000	10 (25.6)
1,000–3,000	18 (46.2)
3,001–5,000	8 (20.5)
>5,000	3 (7.7)
**Licensed bed count**	
1–300	22 (56.4)
301–500	14 (35.9)
>500	3 (7.7)
**Teaching hospital**	
Yes	10 (25.6)
No	29 (74.4)
**Contracted cafeteria food service operations**	
Yes	11 (28.2)
No	28 (71.8)

We calculated the mean nutrition composite scores for the overall hospital and for the individual HNES sections (ie, cafeteria, vending machines, and gift shops) in absolute numbers and in percentages of the total possible points (Table 2). The overall nutrition composite score was a combination of the 3 individual sections, and for this score, hospitals averaged less than 25% of the total possible points. On average, hospitals scored a higher percentage on the vending machines section than on the cafeteria or gift shop sections. The mean gift shop score was below zero because of the numerous deductions made for unhealthful food and beverage options.

**Table 2:** Overall and Sectional Nutrition Composite Scores^a^ of Selected Southern California Hospitals, Hospital Nutrition Environment Scan for Cafeterias, Vending Machines, and Gift Shops (HNES) (N = 39)

HNES Section	Mean Score in Points (SD) of Hospitals Surveyed	Range in Points	Total Possible Points	Percentage of Total Possible Points
Cafeteria	24.2 (8.2)	5.0–41.0	85	28.8%
Vending machines	19.6 (10.6)	2.0–41.0	60	32.7%
Gift shops	−0.4 (2.3)	−6.0–4.0	32	<1%
Overall	43.4 (15.1)	11.0–73.0	177	24.5%

a High nutrition composite scores indicate a favorable consumer nutrition environment.

No relationship was detected between hospital bed size and the overall or individual-section nutrition composite scores. There was also no association between contracted food service operations and the overall or individual-section nutrition composite scores. No significant relationship was detected between overall nutrition composite score and the median income of the hospital neighborhood. In addition, there was no significant relationship between overall nutrition composite score and the education status of people living in the hospital neighborhood.

Our assessment of the prevalence of healthful practices in hospital cafeterias by facility size and by contracted operations (Table 3) showed that almost all of the facilities sold fresh fruit in their cafeterias; however, only about half of the facilities offered a nonfried vegetable on their hot entrée line. Fewer than half of the cafeterias designated most shelf space for healthful items such as low-sugar cereals, baked chips, and 100% juice, and only 15% of cafeterias kept unhealthful items away from the point of purchase.

**Table 3:** Results for Cafeteria Section Questions, by Hospital Size and by Food Service Administration Type, Hospital Nutrition Environment Scan for Cafeterias, Vending Machines, and Gift Shops (HNES)

HNES Section Question	All Hospitals	Hospital Licensed Bed Count		Contracted Food service Operations	
	N = 39, n (%)	<300 (n = 22), n (%)	>300 (n = 17), n (%)	No (n = 11), n (%)	Yes (n = 28), n (%)
**Facilitators/barriers**					
Displays signs that encourage general healthy eating	29 (74.4)	14 (63.6)	15 (88.2)	19 (67.8)	10 (90.9)
Has icons that identify specific healthy items on the menu	24 (61.5)	10 (45.5)^a^	14 (82.4)^a^	15 (53.6)	9 (81.8)
Displays signs encouraging healthy choices as part of a wellness program	7 (17.9)	4 (18.2)	3 (17.6)	5 (17.9)	2 (18.2)
Has promotions or pricing strategies for healthy items	3 (7.7)	2 (9.1)	1 (5.9)	2 (7.1)	1 (9.1)
**Grab and Go items**					
Sells fresh fruit	37 (94.9)	20 (90.9)	17 (100.0)	27 (96.4)	10 (90.1)
**Shelf space**					
>50% of cereals contain low sugar	9 (23.1)	6 (27.3)	3 (17.6)	8 (28.6)	1 (9.1)
>50% of chips are baked	8 (20.5)	5 (22.7)	3 (17.6)	5 (17.9)	3 (27.3)
>50% of milk is low-fat/nonfat	16 (41.0)	9 (40.9)	7 (41.2)	13 (46.4)	3 (27.3)
>50% of soda is diet soda	9 (23.1)	6 (27.3)	3 (17.6)	6 (21.4)	3 (27.3)
>50% of juices are 100% juice	17 (43.6)	10 (45.5)	7 (41.2)	11 (39.3)	6 (54.5)
**Beverages**					
Places bottled water at eye level	16 (41.0)	7 (31.8)	9 (52.9)	12 (42.9)	4 (36.4)
Sells bottled water for less than other beverages	16 (41.0)	7 (31.8)	9 (52.9)	13 (46.4)	3 (27.3)
Provides free access to water	38 (97.4)	21 (95.5)	17 (100.0)	27 (96.4)	11 (100.0)
Does not charge for water cups/glasses	26 (66.7)	14 (63.6)	12 (70.6)	20 (71.4)	6 (54.5)
**Menu review**					
Sells at least 1 healthy combo meal	21 (53.8)	9 (40.9)	12 (70.6)	12 (42.9)^a^	9 (81.8)^a^
Sells at least 1 healthy main entrée	35 (89.7)	19 (86.4)	16 (94.1)	24 (85.7)	11 (100)
Sells at least 1 non-fried vegetable	21 (53.8)	12 (54.5)	9 (52.9)	15 (53.6)	6 (54.5)
Sells at least 1 whole grain starch side	16 (41.0)	9 (40.9)	7 (51.2)	13 (46.4)	3 (27.3)
Sells at least 1 noncream–based soup	22 (56.4)	11 (50.0)	11 (64.7)	14 (50.0)	8 (72.7)
Offers a salad bar	27 (69.2)	13 (59.1)	14 (82.4)	17 (60.7)	10 (90.9)
Offers at least 1 low-fat/fat-free salad dressing	27 (69.2)	13 (59.1)	14 (82.4)	18 (64.3)	9 (81.8)
**Point of purchase**					
Identifies items on the menu or in stalls as “healthy” or “light”	25 (64.1)	11 (50.0)^a^	14 (82.4)^a^	16 (57.1)	9 (81.8)
Has any nutrition information posted on the menu boards/brochures	26 (66.7)	12 (54.5)	14 (82.4)	15 (53.6)^b^	11(100)^b^
Has no unhealthful options near the point-of-purchase (eg, cashier)	6 (15.4)	5 (22.7)	1 (5.9)	3 (10.7)	3 (27.3)

a *P* < .05. Significance was set at *P* < .05.

b *P* < .01. Significance was set at *P* < .05.

Large facility size (>300 licensed beds) was significantly associated with having icons indicating a healthful item on cafeteria menu items and with identifying items on the menu or in stalls as being healthful or “light.” Having a contracted food service operation in the cafeteria was significantly associated with having a healthful combination meal available and with having nutrition information posted in the cafeteria. There were no other significant differences detected between healthful cafeteria practices and the facility size or having a contracted food service operation; however, the lack of significance could have been because some of the healthful practices were detected in only a small number of facilities.

## Discussion

This study is the first to use a reliable research instrument to quantitatively describe the consumer nutrition environment of hospital cafeterias, vending machines, and gift shops. Overall, hospitals received a higher percentage score on the vending machine section than on the cafeteria or gift shop section, which indicates that there is more opportunity to improve promotion of health and nutrition in the cafeteria and gift shop venues. Hospitals scored less than 1% of the total possible points in the gift shop section, indicating a particularly poor consumer nutrition environment that warrants better nutrition promotion. Some of the healthful practices observed in the cafeterias were associated with large facility size and with having a contracted food service operation; however, many healthful practices were present in only a small percentage of facilities surveyed. For example, only 7.7% of hospitals surveyed were promoting healthful items with signage in the cafeteria, and only 15.4% of cafeterias had no unhealthful items near the point of purchase. These results indicated that hospitals have many opportunities to improve their consumer nutrition environments and that interventions targeting the consumer nutrition environments of hospital settings are warranted.

A recent study found similar consumer nutrition environments in children’s hospitals across California (21), and Lesser et al concluded that much nutrition intervention is needed to improve the nutrition environments of children’s-hospital cafeterias. Our findings suggest that poor nutrition environments are not exclusive to cafeterias in children’s hospitals. Our study’s findings indicate that cafeterias in hospitals that serve all populations, not just pediatric populations, are in need of interventions to improve their consumer nutrition environments. In addition, hospital vending machines and hospital gift shops also warrant nutrition-improvement interventions.

The lowest sectional nutrition composite scores were found in hospital gift shops. Almost all gift shops surveyed in this study were staffed by volunteers and were managed through volunteer service departments. Because registered dietitians are not typically staffed in volunteer services departments and because these departments are not usually involved in quantity food procurement and preparation, the poor nutrition composite scores from this section were not entirely surprising. Gift shops often serve as a source of revenue for hospitals; thus, items with high profit margins, such as candy and soft drinks, are readily available. Some gift shops, however, did score points for having small serving sizes available (eg, individually wrapped chocolates instead of large boxes of chocolates) and for limiting the unhealthful items available within 5 feet of the point of purchase. Several hospitals also increased the price of unhealthful items to offset a lower price on more healthful items. Such pricing differentials were also observed in both the cafeteria and in the vending machines sections.

Large hospital cafeterias displayed healthful-choice icons on their cafeteria menus more than small hospital cafeterias, and this may be because large hospitals have more financial and human resources than small hospitals and are therefore able to implement menu-labeling programs. Having contracted food service operations was associated with having nutrition information posted in hospital cafeterias, whereas having internally managed food service operations was not. This could be explained by the fact that most contract food service companies have regional or national offices that employ dietitians and other staff members to develop, analyze, and distribute menu and nutrition information to facilities across their associated territories. In contrast, internally operated hospital food service departments typically develop their own cafeteria menus, and these departments may not have the human resources or the sophisticated software programs to perform nutrient analyses on all items served. For this reason, internally operated food service departments could consider using recipes that have already been analyzed, or they could consider temporarily contracting with a registered dietitian to perform recipe analyses for their commonly served items to ensure that nutrition information is readily available to the consumer at the point of purchase.

As a first look at the hospital consumer nutrition environment, this study provides detailed information about potential areas of need and opportunities for improvement. However, the study was underpowered to detect minor differences between nutrition composite scores and facility size or food service operations management. As such, future studies should consider surveying more hospitals to stratify results and detect small-scale differences in consumer nutrition environments. In addition, the HNES and its sections do not currently have specific cut-off points for what constitutes a minimally, moderately, or highly healthful environment. As more facilities implement nutrition strategies and conduct the scan to evaluate intervention results, these cut-off points should be developed for interpretation purposes.

Given the gap in information about consumer nutrition environments in hospitals, the results of our study are the first to give public health practitioners insight into the current state of hospital consumer nutrition environments. Although one recent study did use an adapted NEMS tool to analyze 14 children’s hospital cafeterias (20), the present study was the first to use HNES to analyze a variety of food service operations (ie, cafeterias, vending machines, and gift shops) in a large, diverse sample of hospitals. The results from the current study can be used to inform initiatives aimed at nutrition promotion in this setting and to help hospital managers and administrators identify ways to improve their own facility’s consumer nutrition environment.

The study has several limitations. Given that the sample of hospitals surveyed was a convenience sample from a limited geographic location, the findings may not be generalizable to hospitals across the United States. California is home to a wide variety of produce farms (22), and many California farmers are involved in local sourcing projects. As such, it is possible that the hospitals surveyed in this study may have better access to fresh produce than hospitals in other geographic locations. In addition, most of the hospitals surveyed in this study were in urban or suburban areas. Because of their location, they had fairly easy access to professional and business resources (eg, multiple food service distribution centers, large food procurement companies), which may not be as accessible to hospitals in rural areas.

Ours is the first study to explore and quantitatively analyze multiple consumer nutrition environments of hospitals by using a valid and reliable instrument, HNES. The individual section and overall nutrition composite scores reported in this study establish a baseline of data against which other hospitals and health care systems can compare their own scores. Overall, hospitals typically scored less than one-fourth of the total HNES nutrition composite score points; they also scored less than 1% of possible points in the gift shop section and approximately one-third of possible points on the vending machine section. These data may also be useful in establishing a national database so that ecology-based initiatives know what specific environments should be targeted in the hospital setting. Our hope is that health care systems will use the HNES tool and the data presented in this study to inform their own food service operations and to improve the consumer nutrition environments of hospitals across the United States.
